# Conquering the Tyranny of Number With Digital Microfluidics

**DOI:** 10.3389/fchem.2021.676365

**Published:** 2021-05-26

**Authors:** Ya-Tang Yang, Tsung-Yi Ho

**Affiliations:** ^1^ Department of Electrical Engineering, National Tsing Hua University, Hsinchu, Taiwan; ^2^ Department of Computer Science, National Tsing Hua University, Hsinchu, Taiwan

**Keywords:** digital microfluidics, electrotwetting, microelectronics, electrowetting on dielectric, electrowdewetting

## Abstract

The development of large-scale integration based on soft lithography has ushered a new revolution in microfluidics. This technology, however, relies inherently on pneumatic control of micromechanical valves that require air pressure to operate, while digital microfluidics uses a purely electrical signal on an electrode for droplet manipulation. In this article, we discuss the prospect and current challenges of digital microfluidics to solve the problem of the tyranny of numbers in arbitrary fluidic manipulation. We distill the fundamental physics governing electrowetting and their implications for specifications of the control electronics. We survey existing control electronics in digital microfluidics and detail the improvements needed to realize a low-power, programmable digital microfluidic system. Such an instrument would attract wide interest in both professional and non-professional (hobbyist) communities.

## Introduction

Microfluidics refers to the behavior, precise control, and manipulation of fluids on a small scale (typically sub-millimeter or below). As in other micro technology, the physics of miniaturization is the core principle of microfluidics and often deviates from human ordinary experience at the macro scale (typically meter or above). One prominent example is the dominance of surface tension. As emphasized by Kim, “if we were to live in a submillimeter world, the principal phenomenon that would concern us would be capillary force, not gravity nor inertia. At this scale, we would undoubtedly have invented numerous machines driven by surface tension” (Tabeling, 2005). Surface tension can be viewed as the elastic tendency of a fluid surface to acquire the least surface area possible. Since surface tension was discovered by Laplace (de Gennes et al., 2004) as the curvature of two immiscible fluid phases, it has played a central role in fluidic mechanics. An inviscid theory by Rayleigh explained the capillary instability of a cylindrical column of liquid: a liquid column held together by surface tension ultimately breaks into discrete drops (Rayleigh, 1879). Taylor (1934) formulated a theory of droplet formation based on competition between surface tension and shear forces. The large ratio of surface area to volume typical for microfluidic systems ensures that surface tension profoundly influences fluid behavior (Atencia and Beebe, 2005).

Microfluidics includes digital microfluidics, which is based on electrowetting, and uses capillary force to manipulate microdroplets. The term “electrowetting” was introduced by Beni and Hackwood (1981) and refers to the apparent increase in the wetting on a surface with an electrical field, akin to the concept of electrocapillarity introduced by Lippmann (1875). Electrodewetting is the opposite of electrowetting and makes a liquid appear to wet a surface less than its inherent ability with an applied electrical field.

Recently, Berge (1993) introduced a thin insulator to separate the conductive liquid from the metallic electrodes to eliminate the problem of electrolysis. Digital microfluidics was shown to be capable of not only switching a droplet between beading and wetting but also moving it along desired paths on a surface. In addition, digital microfluidics was shown to generate water droplets from a reservoir as well as move, split, and merge them (Cho et al., 2003). These fundamental microfluidic operations have made lab-on-a-chip applications possible. Subsequently, several pioneering researchers have popularized digital microfluidics or, more generally, manipulation of droplets using electrical signals (Li and Kim, 2000; Pollack et al., 2000; Pollack et al., 2002; Cho et al., 2003). These techniques have been applied in biomedicine, chemistry, and biology (Quiliet and Berge, 2001; Cho and Moon, 2008; Abdelgawad and Wheeler, 2009; Jebrail and Wheeler, 2010; Pollack et al., 2011; Choi et al., 2012) and have even led to commercially available liquid lenses and diagnostic kits (Li and Kim, 2020).

**FIGURE 1:**
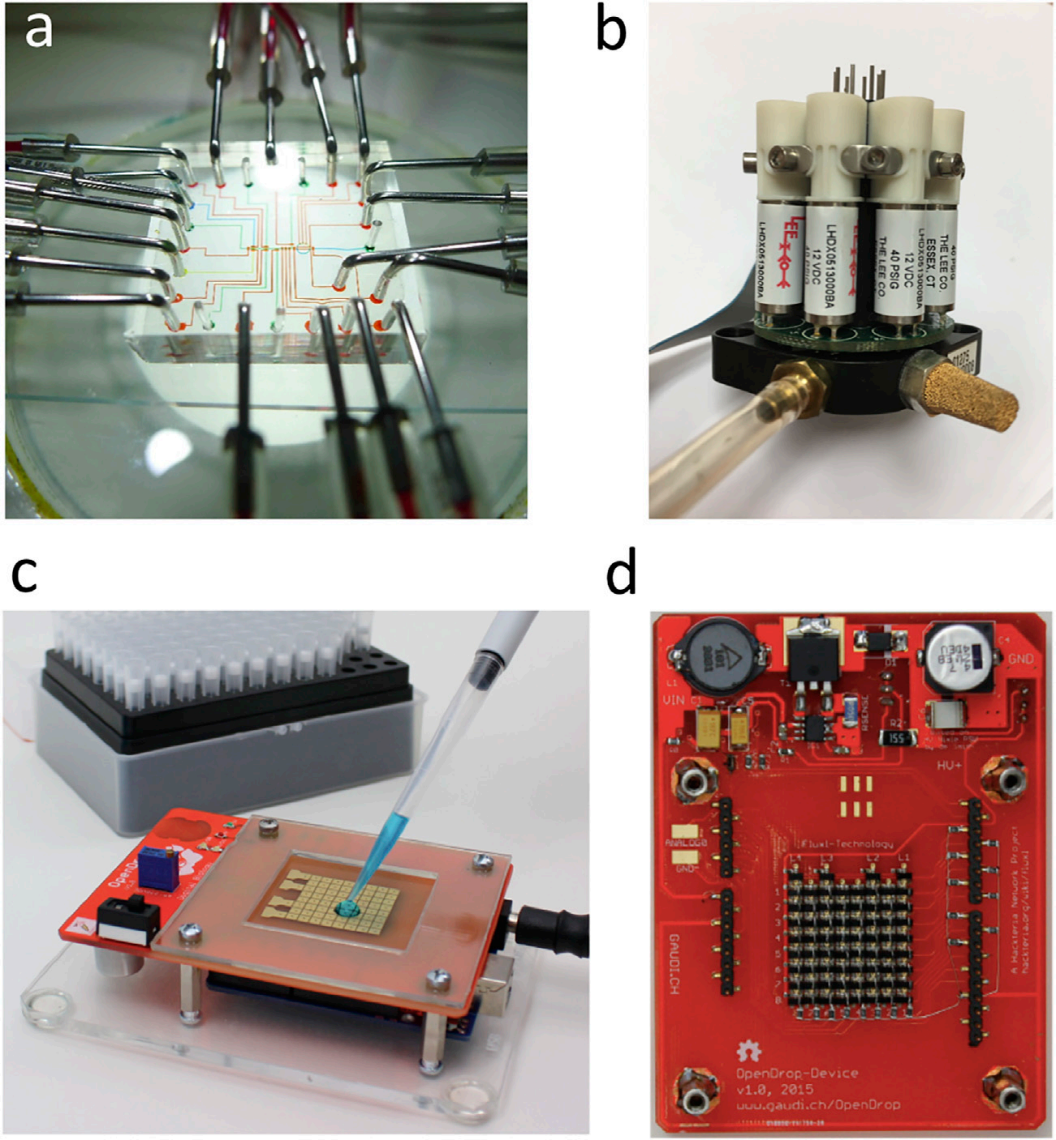
Microfluidics based on soft lithography vs. digital microfluidics. **(A)** Microfluidic chip built with multi-layer soft lithography. Microchannels are colored with food dye. Stainless steel tubes are used to provide the fluid input and pneumatic control. **(B)** Manifold of solenoidal valves used in pneumatically controlled micromechanical membrane valves of a microfluidic chip based on soft lithography (Photo by Ya-Tang Yang). **(C)** OpenDrop is an example of digital microfluidics. OpenDrop consists of an array of electrodes and uses an electrical voltage to manipulate the droplet (Alistar and Gaudenz, 2017). **(D)** Back side of OpenDrop device showing an array of 64 field effect transistors (FETs) (Alistar and Gaudenz, 2017).

The development of discrete components such as vacuum tubes and transistors enabled the development of the first electronic computers. Electrical engineers soon realized that they could design circuits of arbitrary complexity, but that there was a practical limit to the complexity of circuits (Mead and Conway, 1979). They called this limit the “tyranny of numbers,” and the microfluidics community has faced a similar limitation on circuit design (Quake, 2018). Although large-scale integration (LSI) based on soft lithography ushered a new revolution in microfluidics in 2002 (Thorsen et al., 2002), this technology relies inherently on micromechanical valves that require air pressure to operate. It is very difficult to miniaturize a pneumatic control system based on electromagnetic solenoidal valves and pressure sources, and such a system requires a lot of power to operate (Figures 1A,B). Digital microfluidics, however, uses purely electrical signals on an electrode to manipulate droplets and can be seamlessly integrated with existing electronic devices (Figures 1C,D). Given the unparalleled degree of complexity achievable with microelectronics, intimate coupling between microelectronics and digital microfluidics is an obvious route to conquer the tyranny of numbers in microfluidics. There are also subtle conceptual analogies between digital microfluidics and microelectronics. Obviously, the droplet is a discrete vessel that moves. The motion and control can be “digitally” understood by a micro-computer. In microelectronics, Carver Mead began collaborating in 1975 with Lynn Conway from Xerox PARC to use computer software to design an integrated circuit, a method now known as electronic design automation (EDA) (Mead and Conway, 1979). EDA is essential because a modern semiconductor chip can have billions of components. EDA has similarly been used to optimize the routing and design of a digital microfluidics system (Ho et al., 2010). The physics of electrowetting on dielectric (EWOD) also bears many similarities to that of the metal oxide field effect transistor (MOSFET). Both EWOD and the MOSFET use an electric field across a dielectric to exert control. In the MOSFET, the electric field is used to modulate the carrier flow in the conduction channel inside the semi-conductor layer of the device. In EWOD, the electric field is used to apply an actuation force to the droplet.

There are extensive reviews on applications and fundamental physics of digital microfluidics (Quiliet and Berge, 2001; Mugele and Baret, 2005; Cho and Moon, 2008; Abdelgawad and Wheeler, 2009; Jebrail and Wheeler, 2010; Pollack et al., 2011; Choi et al., 2012), and we do not intend to repeat these efforts. Instead we review the subject from the angle of microelectronics and suggest areas on which to focus future work. In particular, we would like to see how associated electronics can be optimally designed for a specific EWOD system, and conversely, which EWOD system is most likely to be widely adopted by electronic engineers.

### General Design Consideration

To be more precise in our discussion, we separate a complete digital microfluidic system into the physico-chemical aspect of the EWOD system and the associated electronics. The physico-chemical aspect of the complete system consists of mainly the liquid of the droplet, dielectrics, conducting electrodes, and the substrate. It also includes addition of surfactant molecules in the droplet, filler liquid, and hydrophobic and lubrication coating on the dielectrics. The associated electronics can be divided into the logic control part, high-voltage driver circuits to the EWOD electrode, and peripheral devices. The logic part can be further divided into a central computing unit (CPU) with memory such as a micro controller chip with embedded memory and the “glue” interface logic. The memory stores software with the algorithm for droplet manipulation. The peripheral devices include information display, input devices such as a keyboard, and environmental controls.

### Physics of EWOD

Before we delve into the electronics of EWOD, it is worthwhile to mention several key physics concepts in digital microfluidics and their implications. First, the Young–Lippmann equation gives the contact angle of a droplet sitting on a solid substrate as

$$\cos\left(\theta_{V}\right)=\cos\left(\theta_{0}\right)+\frac{\varepsilon_{0}\varepsilon_{r}V^{2}}{2d\gamma_{GL}},$$ (1)

where *θ*_*v*_ and *θ*_0_ are the contact angles at applied voltage *V* and zero voltage, respectively; *D* and *ε*_*r*_ are the thickness and relative permittivity of the dielectric layer; *ε*_0_ is the permittivity of the vacuum (Mugele and Baret, 2005); and *γ*_*GL*_ is the surface tension between the gas phase and the liquid phase. The contact angle change is determined by the competition between the surface tension and electrostatic energy of the charging capacitance per unit area. To recast Eq. 1 into another useful form, we can express the voltage necessary to induce a given change in contact angle as being proportional to the square root of *d*/*ε*_*r*_:

$$V=\sqrt{\frac{2d}{\varepsilon_{r}}\left(\frac{\gamma_{GL}}{\varepsilon_{0}}\right)\left(\cos\left(\theta_{v}\right)-\cos\left(\theta_{0}\right)\right)}.$$ (2)

In the literature, *d* can vary from ∼10 μm to ∼100 nm, i.e., two orders of magnitude. In contrast, *ε*_*r*_ is a material property; *ε*_*r*_ ∼ 3 for a typical vacuum-coated material such as silicon nitride, and a maximal *ε*_*r*_ ∼ 100 is reported for barium strontium titanate (Moon et al., 2002). Second, the transport of the droplet is analogous to the motion of droplets on a chemically heterogeneous substrate (Brochard-Wyart, 1989). The dissipation can be either dominated by contact line friction or bulk viscous effects (Mugele and Baret, 2005). In practice, an EWOD system lies between these two extremes. In either case, liquid motion can only be achieved above some threshold voltage. A critical frequency, *f*_*c*_, can be calculated for a given EWOD configuration (Chartterjee et al., 2009). Below this frequency, the force acting on the droplet is electrostatic and arises from charges accumulated near the three-phase contact line. Above the critical frequency, a dielectrophoretic (DEP) force dominates to pull the droplet toward the activated electrode.

### High-Voltage Driver Circuit to an EWOD Electrode

**FIGURE 2:**
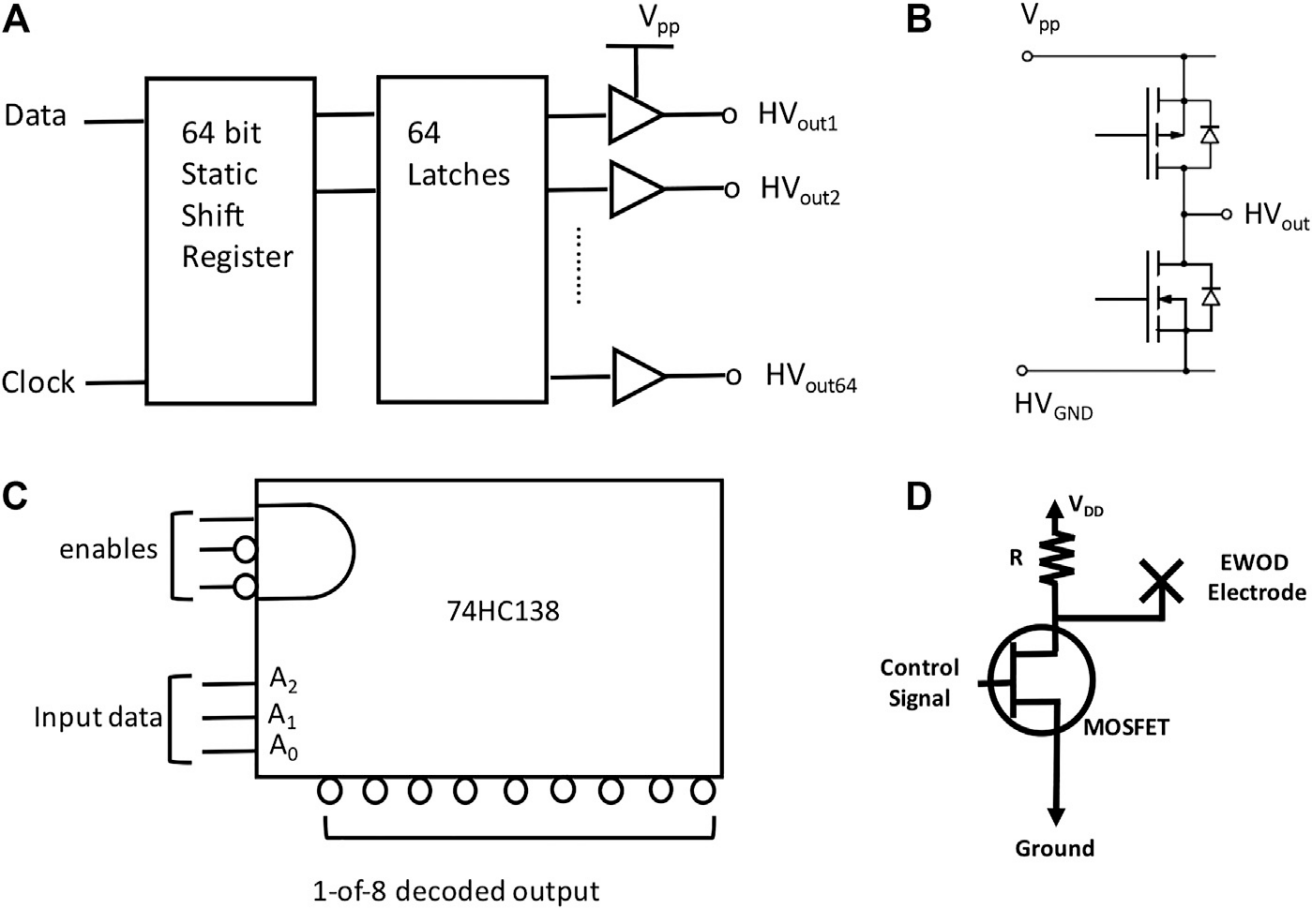
Serial converter and multiplexor. **(A)** Functional diagram of HV507. The HV507 used in one version of OpenDrop (Alistar and Gaudenz, 2017) is a low-voltage serial-to-high-voltage parallel converter with 64 push–pull outputs. The device consists of a 64-bit shift register and 64 latches (Adapted from datasheet of Supertex Inc.). **(B)** Output equivalent of HV507. **(C)** Functional diagram of 74HC138. The 74HC138 accepts a three-bit binary address on input pins A0, A1, and A2 and produces a 1-of-8 decoded output (Horowitz and Hill, 1980). The multiple enable lines allow for the parallel expansion of decoders. **(D)** Switching circuit based on an FET and a resistor load. A control signal is applied to the gate of the metal-oxide FET (MOSFET) for the switching operation (Guo et al., 2021).

The major challenge for EWOD control electronics is to design driver/switching circuits capable of applying high voltage (tens of volts to ∼800 V_rms_) to a large array of EWOD electrodes. The choice of insulating dielectric largely dictates the actuation voltage necessary for droplet motion (Moon et al., 2002). For over a decade since EWOD was first introduced, EWOD users have been stuck with solid state relays and power-thirsty high-voltage amplifiers (Fobel et al., 2013) [For completeness, we mention a side development using a thin-film transistor to provide direct control over EWOD operation (Hadwen et al., 2012). This approach, however, requires expensive fabrication from display technology.] Ironically, high-voltage MOSFETs (HV MOSFETs) have existed for decades but remained elusive to the EWOD community until Gaudi’s laboratory introduced OpenDrop (Figures 1C,D). In designing OpenDrop, Gaudi’s group completely abandoned the solid state relay in favor of the HV MOSFET, which largely reduces the power consumption. To make the discussion on power dissipation more specific, we consider an EWOD electrode connected to the drain of an FET device in Mickey Mouse Logic (M^2^ L) (Horowitz and Hill, 1980) as shown in Figure 2D. Because the energy stored in a capacitor of capacitance *C* and voltage *V* is 1/2 CV^2^, and an equal amount of energy is dissipated by the resistive charging circuit, the power dissipated for a switching frequency *f* is

$$P=V^{2}fC.$$ (3)

In other words, an MOS device consumes power proportional to its switching frequency and the square of the drain voltage *V*. Although this expression is for an FET switch, it also fundamentally limits power consumption from the standpoint of computation (Mead and Conway, 1979). In a practical situation with dc voltage, *f* ∼ 1/Δ*T*, where Δ*T* is the time interval between the actuation sequence. Typically, Δ*T* ∼ 1 s for a 10 μL droplet moving across a milliliter-scale electrode (Guo et al., 2021).

### Choice of Microcontroller and the Need for Interface Logic

The microcontroller was first introduced in the 1970s (Horowitz and Hill, 1980) and is now available at a few dollars per piece with embedded flash memory. It only needs an external quartz crystal as the frequency-determining element of the clock to operate. High clock frequency, over 1 GHz, should be avoided because this will increase the complexity of the printed circuit board layout. The limitation on direct usage of the microcontroller is the programming. A good and intuitive programming language will lower the barrier for users.

We should point out the need for glue logic in between the microcontroller unit and driver circuit. Because a typical powerline ac voltage is in the range of 110–220 V worldwide, integrated circuits such as the HV507, made from high-voltage complementary metal oxide semiconductor (HVCMOS) technology, can handle up to 300 V with 64 channels (Alistar and Gaudenz, 2017) (For reference, Figures 2A,B shows a simplified functional diagram and its output equivalent of HV507.) These ICs, however, use a serial interface with a latched data output because the designer wants to minimize the number of pinouts. The serial interface requires the user to program a well-timed control signal, which could represent a hurdle for the user. A parallel interface with multiplexing allows more intuitive design for users. These interfaces can be easily built with a combination of off-the-shelf multiplexor ICs, for example the 74HC138 from the 74 logic family (Horowitz and Hill, 1980), and an array of discrete MOSFET devices (Figures 2C,D). In the future, application-specific integrated circuits (ASICs) could also integrate multiplexors with HV MOSFET devices.

### Software and Algorithm

Continued growth of EWOD chips depends on advances in chip integration and design-automation tools. Design-automation tools are needed to ensure that EWOD chips are as versatile as the macro-laboratories that they are intended to replace (Ho et al., 2010; Huang et al., 2011). Furthermore, as more bioassays are executed concurrently on an EWOD chip, system integration and design complexity are expected to increase dramatically. There is now a need to deliver the same level of computer-aided design (CAD) support to the EWOD designer that the semiconductor industry today takes for granted. These CAD tools will allow designers and chip users to harness the new technology that is rapidly emerging for integrated biofluidics.

**FIGURE 3:**
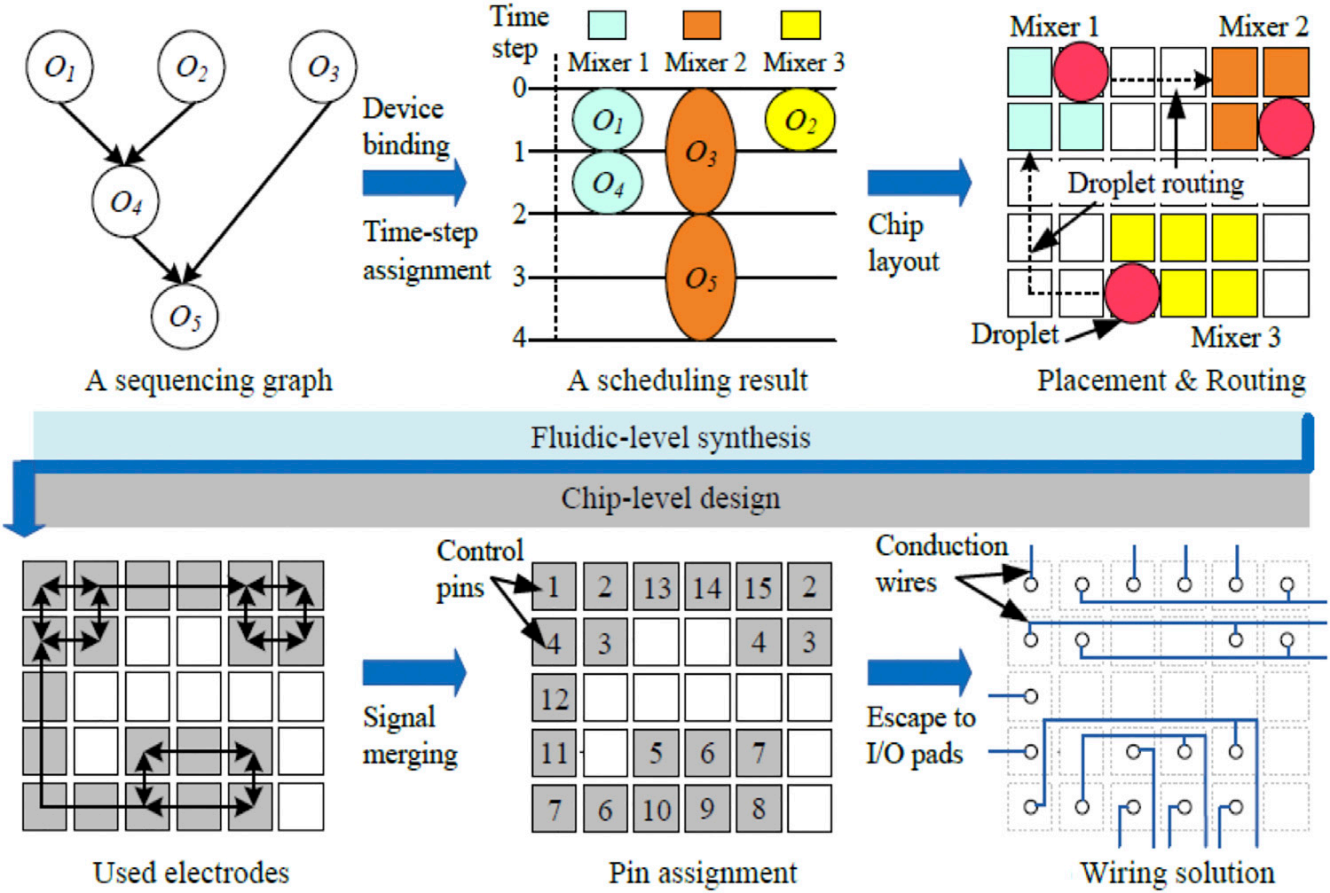
EAD-based design flow of electrowetting-on-dielectric (EWOD) chips. A sequence graph defines the droplet manipulation on an abstract level and subsequently goes through the time-step assignment. The EAD software completes the fluidic-level synthesis by generating the chip layout and proceeds into chip-level design with the pin assignment and wiring solution.

Traditionally, designing and realizing EWOD chips consists of two major stages, fluidic-level synthesis and chip-level design (Chang et al., 2013). In fluidic-level synthesis, different assay operations (mixing, dilution, etc.) and their mutual dependencies are first represented as a sequencing graph, as illustrated in Figure 3. Next, scheduling assigns time-multiplexed steps to these assay operations and binds them to a given number of devices to maximize parallelism. On the basis of the scheduling result, device placement and droplet routing are conducted to generate a chip layout and establish droplet routing connections between devices in a reconfigurable manner. However, chip-level design determines the required control pins and corresponding wiring connections for the underlying electrodes to execute the synthesis result. As illustrated in the lower panel of Figure 3, fluidic-control information on used electrodes is obtained from the previous synthesis. Then, control pins must be minimally and appropriately assigned to electrodes to minimize the bandwidth of input signals. Finally, conduction wires must be routed to establish correspondence between control pins. Reliability is a critical factor in the design flow of EWOD chips and directly affects the execution of bioassays. The problem of trapped charge is the major factor degrading chip reliability. An algorithm has been proposed that not only minimizes the reliability problem induced by signal merging, but also prevents the operational failure caused by inappropriate addressing results (Yeh et al., 2014).

### Prevention of Droplet Evaporation

One important peripheral is the humidified environment to prevent droplet evaporation. Evaporation of the droplet is a serious problem for long-term operation in digital microfluidics. The evaporation of a droplet of diameter d at time t is governed by the law $d^{2}=d_{0}^{2}-\beta t$, where *β* depends on the heat of vaporization and thermal conductivity of the liquid but is independent of droplet size (Middleman, 1998; Tabeling, 2005). The time *τ* for the droplet to disappear is thus

$$\tau =\frac{d_{0}^{2}}{\beta }.$$ (4)

This expression gives the scaling law for evaporation. If the droplet volume shrinks by three orders of magnitude, e.g., from 1 μL to 1 nL, the evaporation time is shortened by a factor of ∼100. Interestingly, this issue of the humidified environment has only recently been tackled electronically with an ultrasonic piezo atomizer (Guo et al., 2021).

### Electrodewetting as an Emerging Disruptive Solution

The recent work on electrodewetting with ionic surfactants raises the possibility of integration with low-voltage complementary metal oxide semiconductor (CMOS) electronics. The EWOD system consists of heavily doped silicon as a conducting electrode and a hydrophilic native oxide of thickness ∼3 nm as the dielectric layer. In contrast to a typical EWOD, a tunneling current flows through the native oxide as electrodewetting happens. The voltage drives the surfactant molecules electrophoretically to induce the change in contact angle and transport of the droplet. Robust and highly durable electrodewetting can be switched for over 10^4^ cycles. In comparison, dielectric charging would degrade the performance of an EWOD device after just a few hundred cycles in air. The detailed physics of such electrodewetting is still largely unexplored at the time this article is written (de Gennes et al., 2004). Because this work demonstrates electrodewetting with a large class of liquids and surfactants, we surmise that the same piece of electrodewetting physics can be readily transferred to an EWOD system based on non-silicon substrates.

### Functional EWOD Dielectrics

Even though this paper primarily focuses on electronics, we should point out the singular importance of EWOD dielectrics because this dielectric layer directly touches the droplet. Ideally, the dielectric layer needs to be thin and have a high dielectric constant and breakdown voltage. It also needs to be hydrophobic and slippery to provide lubrication for the droplet motion. Moreover, it needs to be engineered to be anti-foul to minimize adverse contamination. Even though many materials such as silicone, photoresist, silicon nitride, and silicon dioxide have been used, one can never exhaust all dielectric materials. For example, as the semiconductor industry is squeezing every bit of improvement out of silicon MOS technology, it endows us with a rich repertoire of materials for EWOD to leverage. A high-dielectric-constant material such as barium strontium titanate (BST) is equally applicable to MOS and EWOD (Moon et al., 2002). Although do-it-yourself limits the choice of materials to more readily available ones such as food wrap, we argue that a highly functional yet producible material should be used even though it requires specialized equipment.

## Conclusion

While digital microfluidics still needs much improvement to become widely adopted in non-academic communities, our intention was to articulate the requirements from the “electronics” point of view to show that digital devices are becoming ever more cost-effective and user-friendly. In particular, we should point out a very promising trend: the cost for an EWOD control system has been reduced by about two orders of magnitude from ∼USD$10,000 to ∼USD$100 (Fobel et al., 2013; Alistar and Gaudenz, 2017; Guo et al., 2021). Although a recently reported education kit costing ∼USD$100 is very primitive, EWOD can indeed be available at a very affordable price. In the future, we envision digital microfluidics systems will prevail and merge into the repertoire of electronics modules available for engineers. This will largely come from continuous improvements in reliability and power consumption. Now, approximately 30 years after Berge’s EWOD and with most of the fundamental patents expired, we are starting to see a surge of new companies. Understanding and learning how to design and manufacture more reliable products has increased the chances of harvesting the full potential of EWOD and digital microfluidics. We should also draw the reader’s attention to the “Community Microfluidics” collections, which aim to bring together different DIY microfluidics projects beyond, but including, digital microfluidics technologies (Kong et al., 2017). We expect the digital microfluidics community to grow and offer increasingly versatile designs.

## Data Availability

The raw data supporting the conclusions of this article will be made available by the authors, without undue reservation.
